# Analysis of Swine Conventional Dendritic Cells, DEC205^+^CD172a^+/−^CADM1^+^, from Blood and Spleen in Response to PRRSV and PEDV

**DOI:** 10.3390/v11111001

**Published:** 2019-10-31

**Authors:** Héctor Parra-Sánchez, Lorena Bustamante-Córdova, Mónica Reséndiz, Verónica Mata-Haro, Araceli Pinelli-Saavedra, Jesús Hernández

**Affiliations:** Laboratorio de Inmunología, Centro de Investigación en Alimentación y Desarrollo, A.C., Kilómetro 0.6 carretera la Victoria, Hermosillo 83304, Sonora, Mexico; parraqh@gmail.com (H.P.-S.); lorebcw@gmail.com (L.B.-C.); mresendiz@ciad.mx (M.R.); vmata@ciad.mx (V.M.-H.); pinelli@ciad.mx (A.P.-S.)

**Keywords:** dendritic cells, cDC, DEC205, PRRSV, PEDV, IL-12, naïve T cells

## Abstract

Conventional dendritic cells (cDCs) cannot be infected by porcine reproductive and respiratory syndrome virus (PRRSV) but respond to infection via cytokine production, indicating a possible role in initiation/regulation of the immune response against PRRSV. In this work, we evaluated the responses of splenic and blood cDCs, with DEC205^+^CADM1^+^CD172a^+/−^ phenotype, as well as those of CD163^+^ cells against PRRSV and porcine epidemic diarrhea virus (PEDV). Both populations were incubated in the presence of PRRSV or PEDV with and without naïve CD3^+^ T cells, and cytokine responses were evaluated by qPCR and ELISA. Our results showed that cDCs, but not CD163^+^ cells, produced IL-12 in response to PRRSV. PEDV did not induce IL-12 production. Cocultures of cDCs and autologous naïve CD3^+^ cells resulted in decreased IL-12 production and low expression of IFN-γ transcripts in response to PRRSV. Interestingly, cDCs increased the proliferation of naïve T cells in the presence of PRRSV compared with that achieved with monocytes and peripheral blood mononuclear cells (PBMCs). Cocultures of CD163^+^ cells induced IL-10 and IL-4 expression in the presence of PRRSV and PEDV, respectively. In conclusion, cDCs can selectively produce IL-12 in response to PRRSV but poorly participate in the activation of naïve T cells.

## 1. Introduction

Dendritic cells (DCs) are essential for eliminating pathogens and activating naïve T cells. DCs have different pattern recognition receptors that recognize pathogen-associated molecular patterns (PAMPs), which allows them to detect different types of antigens at the main entry sites, such as the skin [1]. DCs are distributed throughout the body but are especially abundant in sites with high antigen exposure, such as the skin, where they capture, process and, finally, present large amounts of antigens to naïve T cells in the regional lymph nodes [2]. In addition, DCs play a fundamental role in regulating the production of antibodies by B lymphocytes [3] and in transferring antigens to naïve B cells [4]. DCs are also involved in the activation of natural killer (NK) cells as well as their production of interferon (IFN)-γ [5]. DCs play an essential role in the elimination of bacteria and other pathogens, activating both innate and adaptive responses [6]. DCs are divided into two main populations: plasmacytoid DCs, which are the main producers of IFN type 1, and conventional DCs (cDCs). These populations are further divided into two subsets, cDC1 and cDC2. The cDC1 subpopulation has the ability to cross-present antigens to CD8 T cells and differentiate to Th1 cells, while the cDC2 subtype activates CD4 T cells and promotes differentiation to Th2 and Th17 cells [7].

There is continuous interest in studying DCs and their role in the induction and modulation of the immune response against different antigens, such as viruses [8]. In the pig, the main studies to determine the interactions between DCs and viruses have been carried out with monocyte- and bone marrow-derived DCs (moDCs and bmDCs, respectively). These cells have been very useful for understanding the participation of DCs in the immune system. The production of IFN-α by moDCs is inhibited during acute infection with foot and mouth disease virus [9]. On the other hand, moDCs are infected with different strains of porcine reproductive and respiratory syndrome virus (PRRSV) without altering major histocompatibility class II (MHCII) expression [10]. However, the expression of CD80/86 and MHCII is affected by PRRSV infection, and the expression of interleukin-10 (IL-10) is increased [11]. Finally, in vivo, cDCs from PRRSV-experimentally infected pigs are not susceptible to the virus [12,13], while moDCs or bmDCs are susceptible [11]. These discrepancies show that moDCs and bmDCs, despite being an excellent study model, show limitations in understanding the interaction between PRRSV and DCs.

Few studies have evaluated the interaction of swine *bona fide* cDCs and their responses to different viruses, mainly PRRSV and influenza virus. It is known that PRRSV infects monocytes, macrophages [11,14] and moDCs or bmDCs [14]. Nevertheless, lung, trachea and tonsil cDCs are refractory to PRRSV infection [12,13,15]. In the trachea, subtypes of cDCs have a variable response to PRRSV, as cDC1 expresses IFNα, and cDC2 expresses IL-10 [15]. Tonsil cDCs express IL-12 on day 5 of infection [13], and lung cDC1 produces a Th1 cytokine response [12]. In response to influenza virus, lung cDCs, both subtypes cDC1 and cDC2, have the ability to migrate and activate T lymphocytes to differentiate them into Th1 and Th2, respectively [15]. Few studies have investigated the interaction between porcine epidemic diarrhea virus (PEDV) and DCs. One study reported that intestinal DCs promote IL-12 and IFNγ production, whereas activated moDCs stimulate high amounts of T lymphocytes [16]. On the other hand, Peyer’s patch DCs were demonstrated to produce IL-1 and IL-4 and to also induce the proliferation of T lymphocytes; however, the *bona fide* origin of DCs was not evaluated [17]. The main challenge of this study type is to correctly identify the cDCs and/or different subtypes of DCs.

Due to the difficulty of characterizing cDCs in pigs, different strategies have been used for their identification. Skin cDCs have been characterized by the phenotype CD172a^neg^XCR1^pos^ for cDC1 and CD172a^pos^XCR1^neg^ for cDC2 [18]. In the lungs, cDC1 and cDC2 were described as CD172a^neg^XCR1^pos^ and CD163^neg^CD172a^pos^XCR1^neg^, respectively [19]. In blood, cDCs were characterized as CD135^+^CD14^-^CD172a^low^CADM1^+^wCD11R1^+^ and CD135^+^CD14^-^CD172a^+^CADM1^+^CD115^+^wCD11R1^+^CD1^+^ for cDC1 and cDC2, respectively [20]. Recently, our laboratory characterized the cDC subtypes in lymphoid tissues [21] as MHCII^high^CADM1^high^CD172a^−/low^ for cDC1 and MHCII^high^CADM1^high^CD172a^+^ for cDC2; we also characterized the cDC subtypes in the trachea [15] as CADM1^high^CD172a^−/low^ and CADM1^high^CD172a^+^ for cDC1 and cDC2, respectively. Despite the advances in the characterization of cDCs in pigs, challenges remain due to the lack of specific markers that allow simple identification of these populations.

In lymphoid tissues and blood, the cDC subpopulations express the DEC205 receptor [20,21], which could be key for their identification. The DEC205 receptor is a member of a type I C-type lectin receptor family that has ten C-type lectin domains. This receptor has drawn attention because it participates in the immune response [22] in cattle [23], mice [24] and humans [25], and DCs in these animals reportedly express high concentrations of this receptor. In our laboratory, the swine DEC205 receptor was previously characterized [26], and as mentioned above, its expression was demonstrated in the cDCs of blood and lymphoid tissues.

To date, little is known about how the cDCs of different tissues act against viruses and how these cells promote the activation or differentiation of T lymphocytes. The objective of this work was to characterize spleen and blood cDCs and to evaluate how they respond to PRRSV and PEDV in both the presence and absence of autologous naïve T lymphocytes (CD3^+^ cells). The results show that the use of DEC205 in combination with CADM1 and CD172a permits the identification and evaluation of cDCs. These cells were incubated with PRRSV or PEDV, and cDCs were shown to be efficient producers of IL-12. Despite that the production of this cytokine decreased when the cells come into contact with autologous naïve T lymphocytes, cDCs were still capable of stimulating T lymphocyte proliferation.

## 2. Materials and Methods

### 2.1. Animals

Conventional pigs aged 2 to 4 months that were PRRSV-, PEDV- and influenza virus-free were used. The animals were kept at the facilities of the Centro de Investigación en Alimentación y Desarrollo, A.C. (CIAD, A.C.) and had *ad libitum* access to water and food. The animals were euthanized according to the ethical standards of the Mexican Official Norm NOM-033-ZOO-1995. And the experiment is approved by CIAD, A.C. under Identification code: CE/021-A/2014 (14 April 2015).

### 2.2. Viruses

PRRSV-2 (strain CIAD008; GenBank accession no. DQ250071.1) was propagated in MARC-145 cells in DMEM (GIBCO, Grand Island, NY, USA) supplemented with 10% heat-inactivated fetal bovine serum (FBS; GIBCO, Grand Island, NY, USA), 100 IU penicillin mL^−1^ and 100 μg streptomycin mL^-1^ (Sigma, St Louis, MO, USA) (complete DMEM). When the characteristic cytopathic effect was observed, the cell cultures were freeze-thawed twice, and the cell lysates were centrifuged at 650× *g* for 20 min at 4 °C. The supernatant containing the virus was collected, titrated, and stored at −70 °C. Dr. Francisco Rivera Benítez kindly provided PEDV from the Centro Nacional de Investigación en Microbiología Animal, INIFAP. PEDV was propagated in Vero cells and the supernatant containing the virus was collected, titrated, and stored at −70 °C [27]. Uninfected MARK-145 and Vero cells received the same treatment than infected cells and were used as mock control.

### 2.3. Collection of Blood and Tissue cDCs

Peripheral blood mononuclear cells (PBMCs) were obtained from blood using tubes containing EDTA (Becton-Dickinson, BD, Bergen County, NJ, USA) and overlaid on a Ficoll-Paque PLUS density gradient (GE Healthcare, Chicago, IL, USA). To obtain blood cDCs, PBMCs were blocked with 10% bovine serum, and monocytes were removed using CD14 microBeads (MACS Miltenyi Biotec, San Diego, CA, North America) according to the manufacturer’s instructions. Subsequently, B cells were eliminated by labeling with anti-CD21 (IgG1, clone BB6-11C9.6; Southern Biotech, Birmingham, AL, USA) and MACS anti-mouse IgG microbeads (Miltenyi Biotec, Bergisch Gladbach, Germany), and LS columns were used according to the supplier recommendations. The resulting cells, free of monocytes and B cells, were used to sort cDCs.

After euthanasia, the spleen was collected and placed in a 50 mL Falcon tube with 30 mL of cold sterile phosphate-buffered saline (PBS) supplemented with 50 μg/mL gentamicin (Gibco, Big Cabin, OK, USA). In a sterile environment, the spleen was washed three times with PBS containing gentamicin and macerated in its entirety with a 100-μm nylon cell strainer and a syringe plunger. The cells were then collected in 50 mL of RPMI 1640 medium (Thermo Fisher, Waltham, MA, USA) supplemented with 2 mM EDTA, 50 μg/mL gentamicin and penicillin-streptomycin (100 units/mL and 100 μg/mL, respectively) (Sigma, USA) and amphotericin B (1.25 μg/mL) (Sigma, USA). Finally, the cells were centrifuged at 1600 rpm for 10 min at 25 °C, and the viability was evaluated with trypan blue exclusion stain. Erythrocytes, if present, were lysed with lysis buffer (10 mM NaHCO_3_, 155 mM NH_4_Cl, and 10 mM EDTA) and washed with RPMI medium.

### 2.4. Flow Cytometry and Cell Sorting

Monocyte- and B cell-free PBMCs were labeled with anti-CD3 (IgG1, clone 145-2C11; Southern Biotech, USA), anti-DEC205 (mice x swine hybrid recombinant antibody, produced in our laboratory), anti-CADM1 (IgY, clone 3E1; MBL, Japan) and anti-CD172a (IgG2b, clone 742215A; Monoclonal Antibody Center, USA). As secondary antibodies, anti-IgG1 PE (CAT BioLegend, USA), anti-FITC swine (6050-02, Southern Biotech, USA), anti-IgG2b Alexa Fluor 647 (Cat No. 1090-31; Southern Biotech, USA) and anti-IgY biotin (Cat No. 610008; Southern Biotech, USA) were used. Finally, streptavidin BV421 (Cat No. 405226; BioLegend, San Diego, CA, USA) was added. All samples were incubated for 15 min at 25 °C, washed twice with PBS supplemented with 1% FBS (PBS-FBS), and centrifuged at 1600 rpm for 10 min. For spleen cells, a previously described protocol was followed; however, CD14^+^ and CD21^+^ cells were not removed with MACS. In contrast, lineage cells were removed using the antibodies anti-CD21, anti-CD3 and anti-CD163 (IgG1, clone MCA2311; Bio-Rad, Des Plaines, IL, USA) and an anti-IgG1 PE as a secondary antibody. Anti-DEC205, anti-CADM1 and anti-CD172a were used as previously described. Cell acquisition and analyses were carried out on a FACSAria III™ instrument (BD Biosciences, San Jose, CA, USA) using the FACS Diva program. To sort DCs, the population was first selected based on its size (FSC) and complexity (SSC), and doublets were eliminated. Then, the expression of CD3 and DEC205 was evaluated in blood- and spleen-derived cells. The CD3^-^DEC205^+^ population was analyzed for CD172a and CADM1 expression, and these cells were identified as splenic cDCs (DEC205^+^CADM1^+^CD172a^−/+^) and blood cDCs (DEC205^+^CADM1^+^CD172a^low/+^). Sorted cells were collected in RPMI 1640 medium supplemented with 10% FBS (Thermo Fisher, USA).

### 2.5. CD3^+^ and CD163^+^ Cell Sorting

CD3^+^ cells were sorted from blood and spleen using two different strategies. In blood, CD3^+^ cells were sorted from a multicolor staining combination to identify cDCs. After elimination of doublets, CD3 and DEC205 expression was evaluated, and CD3^+^DEC205^-^ cells were sorted. In the spleen, one-color staining was performed in which anti-CD3 followed by an anti-IgG1 PE antibody was used, and CD3^+^ cells were sorted. For the CD163^+^ cells, double staining was performed using anti-CD172a and anti-CD163 followed by the corresponding secondary antibodies labeled with Alexa Fluor 647 and PE. In this case, CD163^+^CD172^+^ cells were sorted. Sorted cells were collected in RPMI 1640 medium supplemented with 10% FBS (Thermo Fisher, USA).

### 2.6. cDC and CD163^+^ Cell Stimulation and Cocultures of cDCs or CD163^+^ Cells with CD3^+^ Cells

cDCs and CD163^+^ were stimulated with PRRSV or PEDV at a multiplicity of infection (moi) of 0.1 for 1 h at 37 °C in RPMI 1640 medium supplemented with 10% FBS and washed to remove the virus. The cDCs or CD163^+^ cells (1 × 10^4^) were cultured in a 96-well plate with the mock (as a negative control), PRRSV or PEDV. After 24 h, the culture supernatant was collected, and the cells were harvested to obtain RNA. All samples were kept at −80 °C until use. For the cocultures of cDCs or CD163^+^ with CD3^+^ cells, the cells were treated with both viruses as previously described. Then, cDCs or CD163^+^ were cocultured with autologous CD3^+^ cells (1 × 10^5^ per well) at a ratio of 1:10 (cDCs or CD163^+^ cells: CD3^+^ cells) and incubated at 37 °C with 5% CO_2_ in RPMI 1640 supplemented with 10% FBS. After 24 h, the culture supernatant was collected, and the cells were harvested to obtain RNA. As a control, CD3^+^ cells without cDCs or CD163^+^ cells were also included.

### 2.7. RNA Extraction and Real-Time PCR

RNA from sorted or cultured cells was extracted with the Arcturus PicoPure RNA Isolation Kit (Thermo Fisher Scientific, Vilnius, Lithuania) according to the manufacturer’s recommendations. Ten nanograms of total RNA was used to amplify the mRNA transcripts using real-time PCR with the SYBR Green RT-PCR One-Step Kit (Agilent, Madison, WI, USA). The amplification protocol was as follows: 50 °C for 30 min and 35 cycles of 94 °C for 30 min and 55 °C for 1 min. The primers used to amplify FLT3, IL-4, TGFB, FoxP3, IL-10, IFN-γ, IFN-α have been previously described by others and us. The results were analyzed with the formula 2^−ΔΔCt^ and are expressed as relative ratios.

### 2.8. ELISA

The production of TNF-α (Invitrogen, Carlsbad, CA, USA) and IL-12 (R&D Systems, Minneapolis, MN, USA) in the culture supernatants were quantified using commercial ELISA kits according to the manufacturers’ recommendations.

### 2.9. Evaluation of CD3^+^ Cell Proliferation in Coculture with cDCs Stimulated with PRRSV

Sorted cDCs, CD14^+^ cells and PBMCs were stimulated with PRRSV at an moi of 0.1 in 96-well plates seeded with 1 × 10^4^ cells per well. They were then incubated for 24 h at 37 °C and washed with RPMI-1640 medium to eliminate the remaining virus. Sorted T cells were labeled with carboxyfluorescein succinimidyl ester (CFSE). Briefly, the cells were stained with CFSE for 15 min (mixing every 5 min), and 1 mL of FBS was then added to eliminate the CFSE excesses. The T cells were centrifuged at 1600 rpm for 10 min at 25 °C, cocultured with the cDCs or monocytes at a ratio of 1:10 for 96 h and analyzed with the BD FACS ARIA III instrument.

### 2.10. Statistical Analysis

All data were evaluated by nonparametric tests. To compare the responses of DCs and CD163^+^ cells in the presence or absence of virus (PRRSV or PEDV), differences between groups were analyzed using the Kruskal-Wallis test, and post hoc multiple comparisons were made with the Benjamini and Hochberg false discovery rate (FDR) method. The same test was used to compare the responses of cocultures of CD3^+^ cells with cDC^+^ or CD163^+^ cells in the presence or absence of virus (PRRSV or PEDV). Since the analysis of IL-10 in response to PEDV was not possible due to the low number of sorted cells, two groups, DCs and CD163^+^ cells in response to PRRSV, were compared by the Mann-Whitney test. In all cases, *p* ≤ 0.05 was considered statistically significant.

## 3. Results

### 3.1. Characterization of Blood and Splenic cDCs

**Figure 1:**
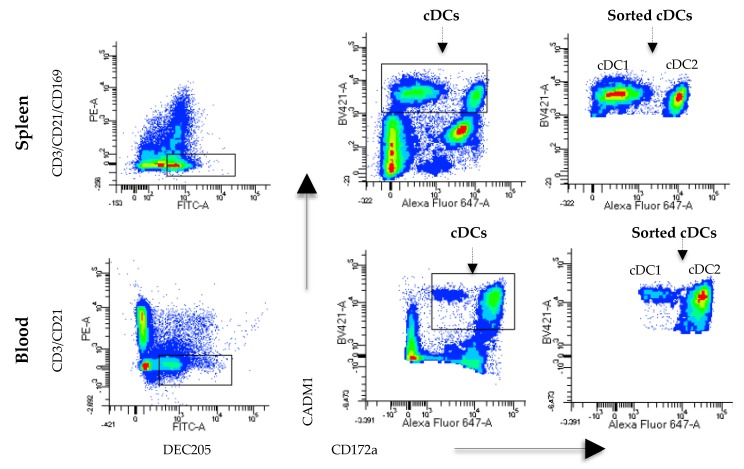
Characterization of blood and tissue conventional dendritic cells (cDCs). To identify the cDCs, the cell population was first selected by size and complexity; then, the doublets were eliminated, and the expression of DEC205 and CD3/CD21 or CD3/CD21/CD163 was evaluated. Then, the expression of CADM1 and CD172a was evaluated in spleen cells and blood. The spleen cDC phenotype was characterized as DEC205^+^CD172a^-/+^CADM1^+^, and blood cDCs were characterized as DEC205^+^CD172a^low/+^CADM1^+^.

Due to the difficulty of characterizing cDCs in pigs, establishing a simple and reproducible strategy for their identification was important. Based on previous reports describing porcine DCs [15,18,19,20,21], we used two strategies to categorize blood and splenic cDCs. As shown in Supplementary Figure S1 and Figure 1, after doublet elimination of the CD14^-^ and CD21^-^free PBMCs, the expression of CD3 and DEC205 was evaluated, and a gate for the DEC205^+^ blood cells was selected. In this population (CD14^-^CD21^-^CD3^-^DEC205^+^), the expression of CD172a and CADM1 was analyzed, and two populations were observed, DEC205^+^CD172a^low^CADM1^+^ and DEC205^+^CD172a^pos^CADM1^+^, which are the supposed cDC1 and cDC2 subsets, respectively, that have been previously described in blood [20]. These two populations were sorted together and named blood cDCs, which were defined as the phenotype DEC205^+^CD172a^low/+^CADM1^+^. To isolate splenic cDCs, after doublet elimination, the cells expressing CD3, CD21 and CD163 were excluded, and DEC205^+^ cells were gated. In this population, we followed the same strategy used for blood cDCs, and two populations were observed, DEC205^+^CD172a^neg^CAMD1^+^ and DEC205^+^CD172a^pos^CAMD1^+^, which are the supposed cDC1 and cDC2 subsets, respectively, that have been previously described in lymphoid tissues [13,21]. Both populations were sorted, and splenic cDCs were defined as DEC205^+^CD172a^−/+^CADM1^+^. To confirm the lineage of these cells, the expression of FLT3 was evaluated by real-time PCR (Supplementary Figure S2). The cells defined as cDCs expressed FLT3, in contrast to the DEC205^+^CD172^-^CAMD1^-^ population. We also evaluated the percentages of the supposed cDC1 and cDC2 subsets in the blood and spleen (Supplementary Figure S3). In agreement with previous reports [20,21], cDC1 prevailed in the spleen, while cDC2 was more predominant in blood.

### 3.2. Splenic cDCs Produce IL-12 in the Presence of PRRSV but not PEDV

**Figure 2:**
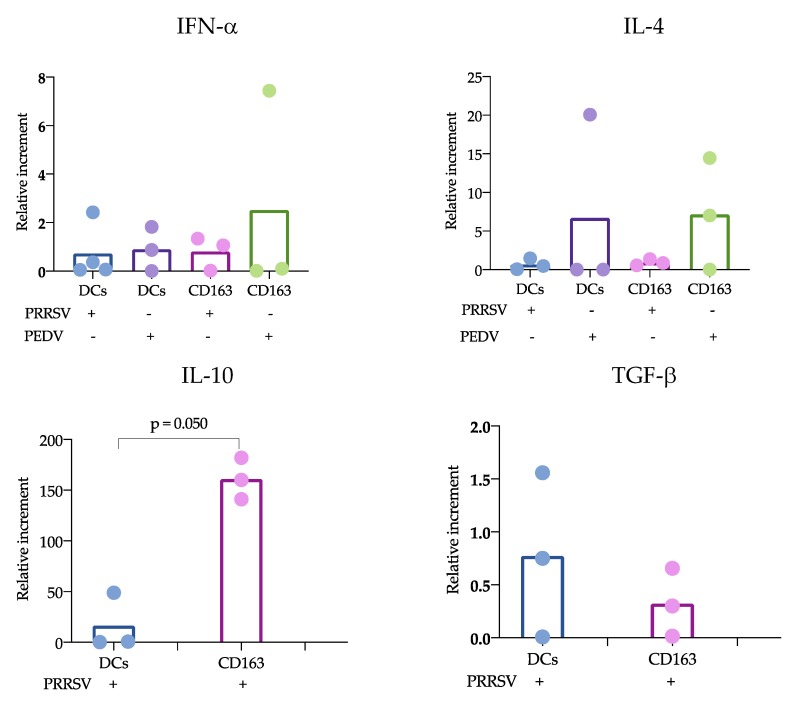
Cytokine expression of splenic cDCs and CD163^+^ cells in response to virus. cDCs and CD163^+^ cells were stimulated with porcine epidemic diarrhea virus (PEDV) or porcine reproductive and respiratory syndrome virus (PRRSV) for 24 h, and cytokine production was analyzed by qPCR. The results were analyzed with the 2^−ΔΔCt^ method and are presented as relative increments. In each figure, the bars represent the mean, and each point represents one pig. Data were analyzed with the nonparametric Kruskal-Wallis test (IFN-α and IL-4) or the Mann-Whitney (IL-10 and TGF-β) test. Significant differences are indicated.

**Figure 3:**
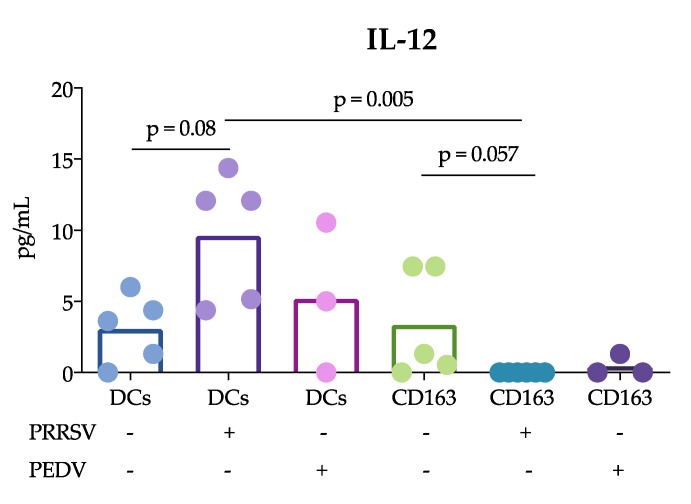

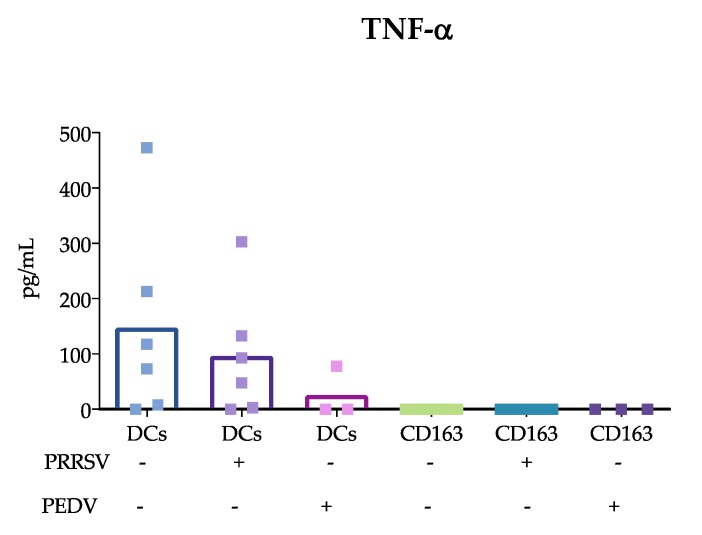
Cytokine production in splenic cDCs and CD163^+^ cells in response to virus. cDCs and CD163^+^ cells were stimulated with PEDV or PRRSV for 24 h, and cytokine production was analyzed by ELISA. In each figure, the bars represent the mean and sample size of at least three pigs; each point represents one pig. Data were analyzed with the nonparametric Kruskal-Wallis test, and significant differences between the bars are indicated.

We next aimed to evaluate the response of two types of antigen-presenting cells (APCs), namely, blood and splenic cDCs and CD163^+^ cells; the first were sorted as previously described (Figure 1), and the second were sorted for the phenotype CD163^+^CD172a^+^ (Supplementary Figure S4), which were named “CD163^+^ cells”. According to previous reports [21], lymph node cells with this phenotype include macrophages and monocyte-derived DCs. cDCs and CD163^+^ cells were incubated separately with the viruses PRRSV and PEDV as described in the Materials and Methods section. Figure 2 shows the cytokine responses as evaluated by qPCR (Figure 2) and ELISA (Figure 3). IFN-α and IL-4 expression was evaluated in cDCs and CD163^+^ cells in response to PRRSV and PEDV, while IL-10 and TGF-β were evaluated in cDCs and CD163^+^ cells only in response to PRRSV. No significant differences were observed in the cytokine expression levels measured by qPCR with the exception of IL-10 mRNA expression. In this case, IL-10 was significantly higher in CD163^+^ cells than in cDCs in response to PRRSV (*p* = 0.050).

On the other hand, when measuring cytokine production by ELISA (Figure 3), we observed that splenic cDCs produced more IL-12 in response to PRRSV than CD163^+^ cells (*p* = 0.005), however, cDCs and CD163^+^ stimulated with PEDV did not show any differences in IL-12 production. Interestingly, TNF-α production was not different between cDCs stimulated with PRRSV and PEDV. In the case of CD163^+^ cells, TNF-α was not detected in any of the treatment groups.

### 3.3. Coculture of APCs with CD3^+^ Cells in the Presence of PRRSV or PEDV Altered Cytokine Expression

**Figure 4:**
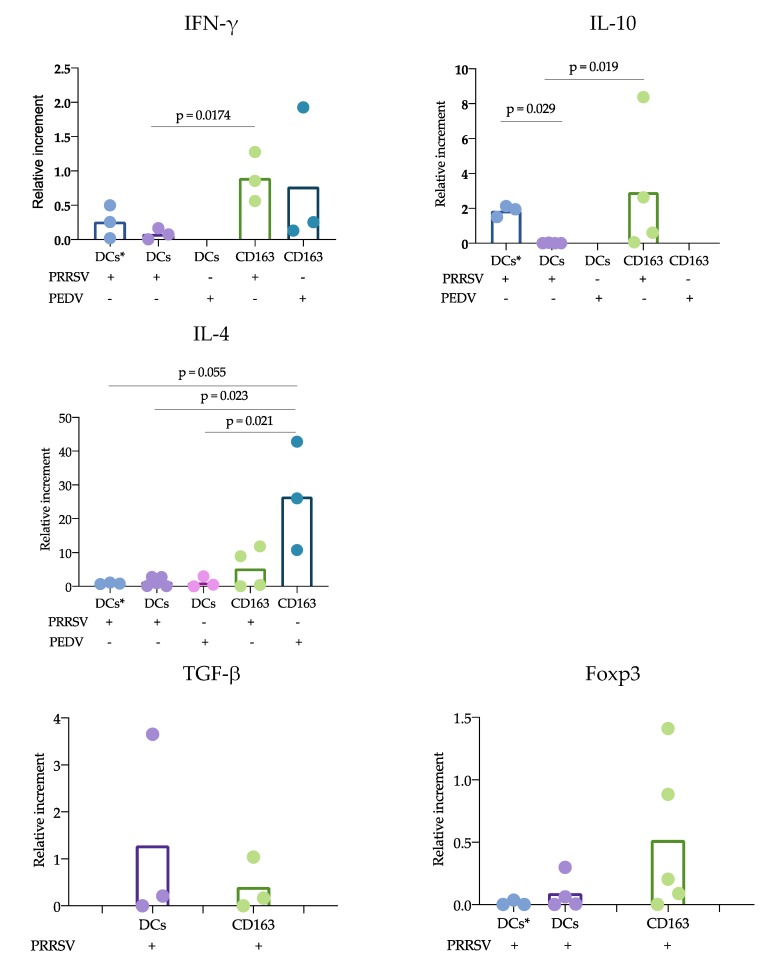
Cytokine expression in cocultures of antigen-presenting cells (APCs) with autologous naïve CD3^+^ cells. Blood cDCs, splenic cDCs or CD163^+^ cells were cocultured with naïve CD3^+^ cells for 24 h, and cytokine expression was evaluated by qPCR. The results were analyzed with the 2^−ΔΔCt^ method and are presented as relative increments. In each figure, the bars represent the mean, and each point represents one pig. Data were analyzed with the nonparametric Kruskal-Wallis test (IFN-γ, IL-10, IL-4 and Foxp3) or the Mann-Whitney (TGF-γ) test. Significant differences are indicated. DCs *, blood cDCs.

To evaluate the effects of coculturing cDCs or CD163^+^ cells with autologous naïve CD3^+^ cells in the presence of PRRSV or PEDV, the APCs were incubated with the viruses for 1 h, followed by the addition of CD3^+^ cells at a 1:10 ratio (APC:CD3^+^ cell) and further incubation for 24 h. Next, the cells were harvested to isolate RNA for further analysis by qPCR, and the supernatant was kept at −80 °C until ELISA analysis. IFN-γ, IL-4, IL-10, TGF-β and Foxp3 were evaluated by qPCR. Significant differences were observed for IFN-γ, IL-4, and IL-10 (Figure 4). In the presence of PRRSV, lower IFN-γ production was observed when splenic DCs were cocultured with CD3^+^ cells than with CD163^+^ cells (*p* = 0.017). Interestingly, no differences were observed in cocultures of blood DCs and CD163^+^ cells. IFN-γ production was not different between CD163^+^ cells stimulated with PRRSV or PEDV. Unfortunately, it was not possible to evaluate the response of cDCs cocultured with CD3^+^ cells in response to PEDV. Cocultures of CD163^+^ and CD3^+^ cells had higher IL-10 expression than splenic cDCs cocultured with CD3^+^ cells (*p*= 0.019). Interestingly, IL-10 expression was higher in blood cDCs cocultured with CD3^+^ cells than in the splenic cDCs cocultures (*p* =0.029). No IL-10 production was observed in splenic cDCs cocultured with naïve CD3^+^ cells in the presence of PRRSV. Analysis of IL-4 showed that this cytokine was expressed at higher levels in the cocultures of CD163^+^ cells and CD3^+^ cells in response to PEDV than in splenic cDCs in response to PEDV (*p* = 0.021) and PRRSV (*p* = 0.023), and a non-significant trend compared to blood cDCs in response to PRRSV (*p* = 0.055). No differences were observed upon comparing the cocultures of CD163^+^ cells and CD3^+^ cells in response to PRRSV and PEDV. No significant differences were observed for TGF-β or Foxp3.

**Figure 5:**
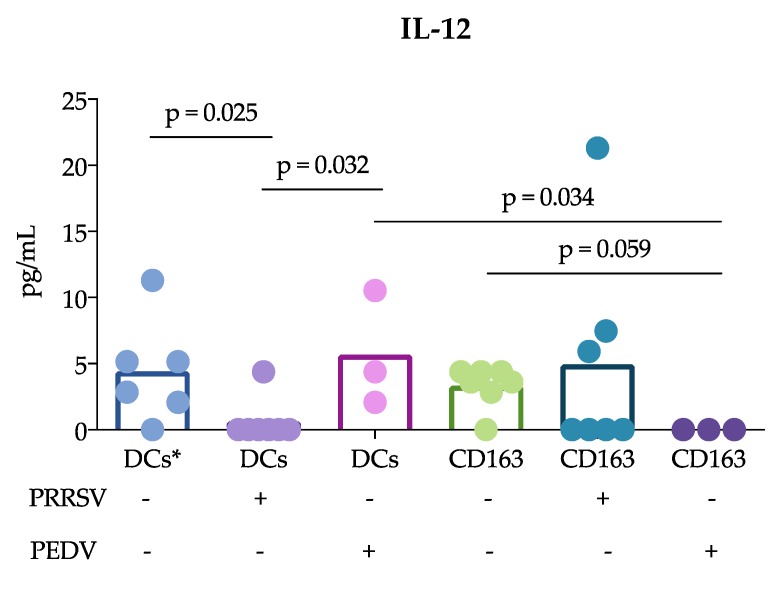
IL-12 production in cocultures of APCs with autologous CD3^+^ cells. Blood cDCs, splenic cDCs and CD163^+^ cells were cocultured with naïve CD3^+^ cells for 24 h, and cytokine production was evaluated by ELISA. The bars represent the mean, and each point represents one pig. Data were analyzed with the nonparametric Kruskal-Wallis test, and significant differences are indicated. DCs *, blood cDCs.

IL-12 was evaluated by ELISA (Figure 5), revealing that PRRSV resulted in lower IL-12 production in the cocultures of cDCs with CD3^+^ cells than in the nonstimulated cocultures (*p* = 0.025) and in those stimulated with PEDV (*p* = 0.032). In contrast, the IL-12 production in the cocultures of CD163^+^ cells with CD3^+^ cells was not different from that in the control and PRRSV-stimulated cocultures. However, PEDV induced significantly lower IL-12 production in the cocultures of CD163^+^ cells with CD3^+^ cells than in the cDC/CD3^+^ cell cocultures (*p* = 0.034).

### 3.4. Proliferation of Autologous T Lymphocytes Stimulated with cDCs Activated with PRRSV

**Figure 6:**
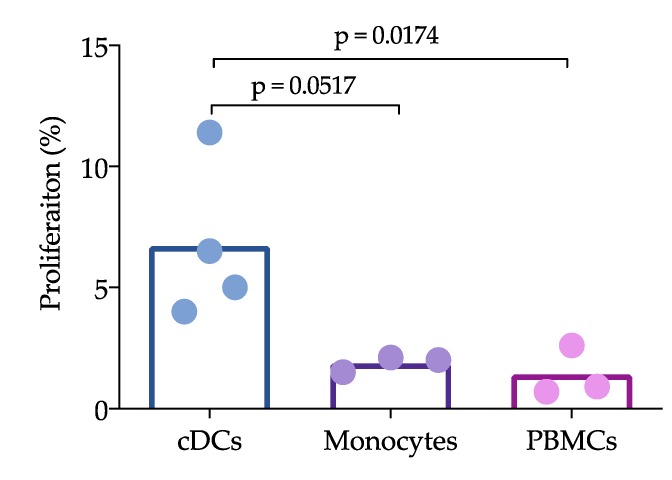
Capacity of cDCs (stimulated with PRRSV) to induce the proliferation of CD3^+^ cells. PRRSV at an moi of 0.1 was used to stimulate the cDCs, monocytes and peripheral blood mononuclear cells (PBMCs) for 1 h. Subsequently, the cells were cocultured with CD3^+^ cells (72 h). Data represent the absolute proliferation, which is the proliferation induced by PRRSV minus the proliferation induced by the mock. The bars represent the mean, and each point represents one pig. Data were analyzed with the nonparametric Kruskal-Wallis test, and significant differences are indicated.

To assess the ability of cDCs to stimulate the proliferation of autologous T lymphocytes, blood cDCs were primed with PRRSV at a moi of 0.1 for 24 h and cocultured with autologous T lymphocytes for 72 h. In addition, monocytes cocultured with autologous CD3^+^ cells and PBMCs stimulated with PRRSV were included. cDCs, monocytes and PBMCs stimulated with the mock were included as controls. Figure 6 shows that cDCs stimulated T lymphocyte proliferation better than PBMCs (*p* = 0.0174), and also better, although borderline significant, than monocytes (*p* = 0.0517) (Figure 6). The proliferation induced by the monocytes was not different from that induced by the PBMCs.

## 4. Discussion

In this work, we used a new and straightforward strategy to identify cDCs using the receptor DEC025. The expression of CD172a and CADM1, used in other studies to characterize DCs [13,15,21], was analyzed in DEC205^+^ cells and CD14^-^ (only in the case of blood cells), CD3^−^, CD21^−^, and CD163^-^negative cells. To confirm the lineages of these cDCs, we analyzed the expression of FLT3 (Supplementary Figure S2), verifying that the selection was adequate because the cells were FLT3^+^. In the spleen, the cDC phenotype was DEC205^+^CD172a^−/+^CADM1^+^, and the subtypes cDC1 and cDC2 in this population were identified as CD172a^-^CADM1^+^ and CD172a^+^CADM1^+^, respectively [21]. However, because cDC1 was characterized as CD172a^low^ in the blood, the CD172a^low/+^ phenotype was chosen for the characterization instead of CD172a^−/+^ [20]. This phenotype is in agreement with previous reports [20,21], and we propose the use of this strategy to facilitate the identification of porcine cDCs with the DEC205^+^CD172a^−/+^CADM1^+^ phenotype in blood and lymphoid tissues.

According to the proposed strategy, blood and splenic cDCs were sorted, and the responses to PRRSV and PEDV were evaluated. Regarding the expression of cytokines, IL-10 was the only one that showed significant differences. IL-10 was produced at significantly higher levels in CD163^+^ cells than in cDCs. While numerous studies agree that PRRSV induces the production of IL-10 in macrophages and moDCs [11,28], limited information is available regarding cDC responses. Our results can confirm that cDCs are poor IL-10-producers in response to PRRSV. Analysis of trachea DCs showed that in response to PRRSV, cDC1 cells expressed IFN-α, while cDC2 cells produced IL-10 [15]; however, others evaluating lung cDCs did not find differences in IL-10 expression. Our results were obtained from splenic cDCs, in which cDC1 predominates over cDC2 (71 ± 3.1 vs 24 ± 3.2 %, respectively); therefore, it is possible that the evaluation of blood cDCs, in which the cDC1 vs cDC2 ratio differs (33 ± 4.3 vs 64 ± 4.3%, respectively), resulted in higher IL-10 production in response to PRRSV compared to that of splenic cDCs. Hence, it is possible that the response of one particular cDC subset to a stimulus, such as PRRSV or PEDV, will depend on the balance of cDC1 vs. cDC2, the amount of antigen used to challenge the cells, and the strain used. The limited results obtained thus far do not allow a complete understanding of the immune response against PRRSV, as no significant differences were observed in IFN-α, IL-4 and TGF-β production, and more studies are needed to verify this hypothesis.

Another important finding of this study was that cDCs were the main producers of IL-12 rather than CD163^+^ cells in response to PRRSV. This result is in agreement with previous reports showing that tonsil cDCs of pigs infected with PRRSV exhibited increased expression of this cytokine for up to 5 dpi [13] and lung cDC1 cells exhibited increased expression for up to 10 dpi [12]. Thus, our results confirm that cDCs are able to produce IL-12 in response to PRRSV in vivo and in vitro. This ability was not observed in CD163^+^ cells or PEDV-stimulated cells. The production of IL-12 is essential for the differentiation of naïve T lymphocytes into Th1 cells [29], and many reports have demonstrated that PRRSV induces IL-12 production [30]. Nonetheless, a correct and early Th1 response against PRRSV was not observed, suggesting that other mechanisms are involved that hinder the effects of IL-12 induced by PRRSV. It is also possible that different PRRSV strains have different potentials to induce IL-12, as reported after the evaluation of type 1 PRRSV strains [12].

Why adaptive protective immune responses against PRRSV are delayed remains an important question regarding PRRSV immunology, and our study proposes some answers to this question. Using the cDC strategy described herein, sorted blood or splenic cDCs were cocultured with CD3^+^ naïve sorted cells, and the responses in the presence of PRRSV and PEDV were evaluated. Our results showed a reduced response of cDCs cocultured with CD3^+^ naïve cells compared with that of CD163^+^ cells cocultures. IFN-γ and IL-10 expression was mainly observed in CD163^+^ cocultures. In this experiment, two sources of cDCs were used, blood and splenic cDCs, which differ in their proportions of cDC1 and cDC2. Interestingly, the blood cDCs cocultures expressed IL-10 at higher levels than the splenic cDCs, and the differences between these cDCs were likely due to the ratio of the cDC1 and cDC2 subtypes. However, the expression of IL-10 in the blood cDCs was lower than that observed in the CD163^+^ cells cocultures, suggesting that the main source of IL-10 in response to PRRSV is CD163^+^ cells. CD163^+^ cells include macrophages and moDCs, both of which are targets of PRRSV infection, in contrast to cDCs, which are refractory. This difference could explain the results and lead to the hypothesis that cDCs poorly participate in the induction of an adaptive immune response against PRRSV. In agreement with this hypothesis, we observed that IL-12 production was reduced in the coculture of naïve T cells with cDCs in the presence of PRRSV (Figure 3). Interestingly, the reduction in IL-12 did not affect the ability of cDCs to induce the proliferation of T cells, which was higher than that induced by whole PBMCs. Unfortunately, we were herein unable to compare the ability of CD163^+^ cells to induce proliferation, but other studies have shown that moDCs are able to induce T cell proliferation [11]. cDCs are not infected by PRRVS, but, apparently, they capture and process the virus to induce proliferation. Furthermore, if the virus is captured, why are these cells not infected? Further experiments with higher moi are needed to evaluate the response of these non-permissive cells, and it is also necessary to determine whether in vitro cDC can capture the virus. One limitation of this work is that the experiments were not designed to evaluate whether cDCs uptake the virus or viral antigens in any way, but rather to characterize spleen and blood cDCs and to evaluate how they respond to PRRSV and PEDV in both the presence and absence of autologous naïve T lymphocytes (CD3^+^ cells); therefore our data cannot provide meaningful biological data on how cDC uptake the virus. However, even considering that this is not the best design, we believe that the data presented here give interesting information to expand our understanding of PRRSV immune response and immunopathology.

There is substantial information to support the notion that cDCs are able to respond to PRRSV with cytokine production, at least of IL-12, IFN-α or IL-10. Additionally, this production can be dependent on the strain used, as FL13 and Lelystad virus do not induce IL-12 [12]. The results obtained in this work show that PRRVS-induced IL-12 production is affected when cDCs are cocultured with naïve T cells. Interestingly, this result suggests that other cytokines or molecules can inhibit IL-12 production and the possible induction of Th1 cells, thus stimulating the polarization to Th2 or Treg cells. Similar results have been reported with the simian immunodeficiency virus. In this case, IL-12 was not produced in the cocultures of DCs with T cells, but the cytokine was produced after stimulation with the TLR7/8 ligand [31]. Another interesting observation was the high IL-4 expression in the CD163^+^ cell cocultures in response to PEDV. In this case, differences were observed when comparing the cocultures of cDCs in the presence and absence of PRRSV and PEDV. IL-4 is an important cytokine for antibody production [17] and has been reported in previous studies evaluating responses to PEDV [17]. One strength of our study was the use of pure cDCs and T cells to evaluate the effects of PRRSV on the possible induction of an immune response; however, one limitation was that the cocultures were maintained for only 24 h, and longer incubation times are required to confirm our hypothesis.

## 5. Conclusions

We herein propose the use of the DEC205 receptor to facilitate the characterization and isolation of cDCs from pig blood and lymphoid tissues. Our results obtained using this strategy showed that cDCs, and no other populations, such as CD163^+^ cells, had the capacity to produce IL-12 when they were in contact with PRRSV. However, the production of both IL-12 and IFN-γ was reduced when cDCs were cocultured with naïve T cells. This result suggests that cDCs poorly participate in the induction of an immune response to PRRSV, at least during the primary response to the infection. This poor participation can be only partially explained because it is not clear how the virus is recognized and internalized for presentation to naïve T cells. These effects can lead to inefficient Th1 cell differentiation and enhanced participation of other DCs, such as moDCs, which produce regulatory cytokines such as IL-10. Additional studies are needed to prove this hypothesis.

## Supplementary Materials

The following are available online at https://www.mdpi.com/1999-4915/11/11/1001/s1, Figure S1: Doublet elimination and fluorescence minus one (FMO) for DEC205 expression, Figure S2: FLT3 relative expression in blood cDCs, Figure S3: Percentages of cDC1 and cDC2 in spleen and pig blood, Figure S4: Purification of the population CD163^+^.
