# Supplementary material for: Analysis of Swine Conventional Dendritic Cells, DEC205+CD172a+/−CADM1+, from Blood and Spleen in Response to PRRSV and PEDV
Source: Viruses. 2019 Oct 31;11(11):1001. doi: 10.3390/v11111001 (PMC6893809; doi:10.3390/v11111001)
Supplement: Supplementary file 1 [file viruses-11-01001-s001.pdf]

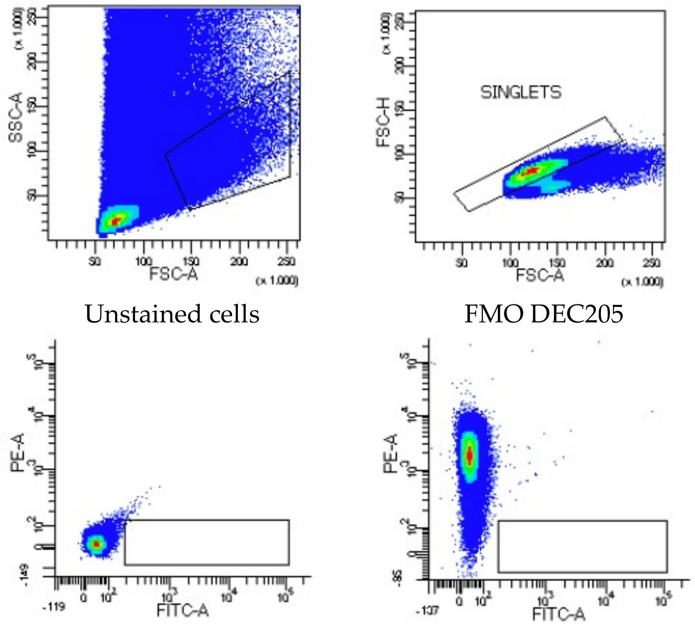

Supplementary 1. Doublet elimination and fluorescence minus one (FMO) for DEC205 expression. A representative experiment to show the controls to identify the cDCs. The cell population was first selected by size and complexity; then, the doublets were eliminated, the unstained cells were analyzed and the FMO for the DEC205 (CD3/CD21/CD163 without DEC205) expression was evaluated.

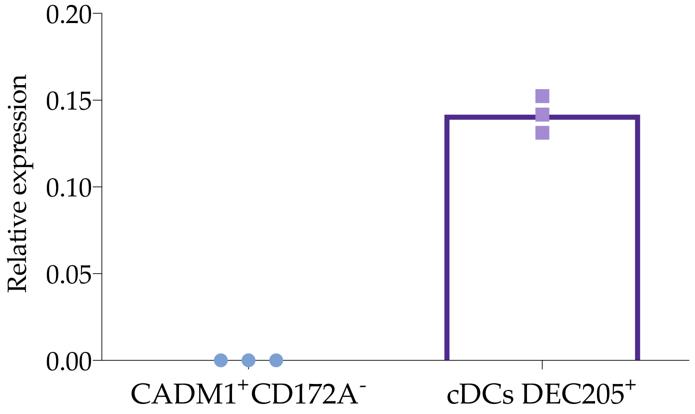

Supplementary 2. FLT3 relative expression in blood cDCs. The expression of FLT3 transcript in blood cDCs was evaluated and the CADM1^{+}CD172a^{-} population was used as a control.

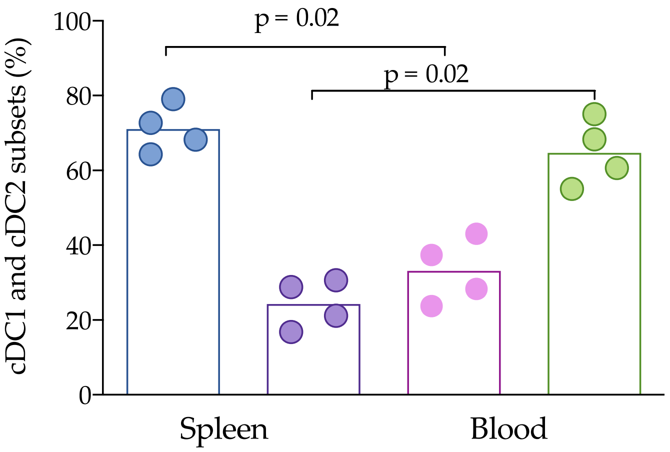

Supplementary 3. Percentages of cDC1 and cDC2 in spleen and pig blood. cDC1 cDC1 and cDC2 were evaluated in spleen and blood; the cDC1 was higher in the spleen while the cDC2 was higher in blood. Data were analyzed with the nonparametric Kruskal-Wallis test and significant differences are indicated.

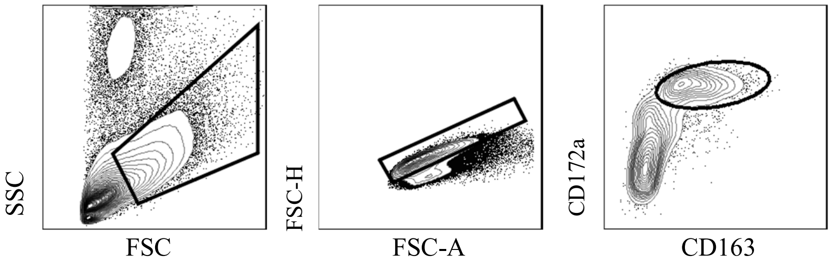

Supplementary 4. Purification of the population CD163⁺. To compare the population of spleen cDCs, the CD163 population was purified through the expression of CD163 and CD172a markers, previously the doublets were eliminated and the population of interest was selected through FSC and SSC.
